# 

**Published:** 1899-02-04

**Authors:** 


					THE HOSPITAL. ? The standard of highest purity."?Lancet, F*b. 4th, 1899.
CADBURY'S M fe* CADBURYS
OOOOA |L JOs. JeK OOOOA
ABSOLUTELY PURE. NO DRUGS OR ALKALIES USED.
'? ^ If X W?S TSJVt Ihflbmajl*
No. 645. Vol. XXV. Satubday,
Feb. 4th, 1899.
? mLti ?iu&izljia& * -
[SubsanptionTo.mB.] palCE TWOPENCE.

				

